# Histocompatibility and Hematopoietic Transplantation in the Zebrafish

**DOI:** 10.1155/2012/282318

**Published:** 2012-06-24

**Authors:** Jill L. O. de Jong, Leonard I. Zon

**Affiliations:** ^1^Section of Hematology-Oncology and Stem Cell Transplant, Department of Pediatrics, The University of Chicago, KCBD 5120, Chicago, IL 60637, USA; ^2^Stem Cell Program and Division of Hematology/Oncology, Children's Hospital Boston-Dana, Farber Cancer Institute, Howard Hughes Medical Institute, Harvard Stem Cell Institute, and Harvard Medical School, Boston, MA 02115, USA

## Abstract

The zebrafish has proven to be an excellent model for human disease, particularly hematopoietic diseases, since these fish make similar types of blood cells as humans and other mammals. The genetic program that regulates the development and differentiation of hematopoietic cells is highly conserved. Hematopoietic stem cells (HSCs) are the source of all the blood cells needed by an organism during its lifetime. Identifying an HSC requires a functional assay, namely, a transplantation assay consisting of multilineage engraftment of a recipient and subsequent serial transplant recipients. In the past decade, several types of hematopoietic transplant assays have been developed in the zebrafish. An understanding of the major histocompatibility complex (MHC) genes in the zebrafish has lagged behind transplantation experiments, limiting the ability to perform unbiased competitive transplantation assays. This paper summarizes the different hematopoietic transplantation experiments performed in the zebrafish, both with and without immunologic matching, and discusses future directions for this powerful experimental model of human blood diseases.

## 1. Introduction

In the past few decades, the zebrafish has emerged as an outstanding vertebrate animal model for studying developmental hematopoiesis (reviewed in [1, 2]). In this same time frame, the understanding of the biology of adult hematopoietic stem cells has also blossomed, predominantly due to hematopoietic transplantation experiments performed in mice (reviewed by Orkin and Zon in [3]). To capitalize on the advantages of the zebrafish model (small size, high fecundity, rapid maturation, external fertilization, and the ability to perform large-scale genetic and chemical screens), a zebrafish hematopoietic transplantation assay was needed. 

Developing a transplantation assay in the zebrafish required a different approach than that used in mice. While differential expression of CD45 isoforms is generally used to distinguish between donor and recipient cells in murine transplant assays, these reagents are not available for zebrafish. Instead, scientists have utilized transgenic technology to make zebrafish expressing green fluorescent protein (GFP) or other fluorochromes under the influence of an ubiquitous or a tissue-specific promoter. These fluorescently labeled donor cells are transplanted into fluorochrome-negative recipients, and engraftment is monitored at various time points after transplant.

## 2. A History of Hematopoietic Transplantation in Zebrafish

### 2.1. Adult Marrow Cells into Embryos

**Figure 1:**
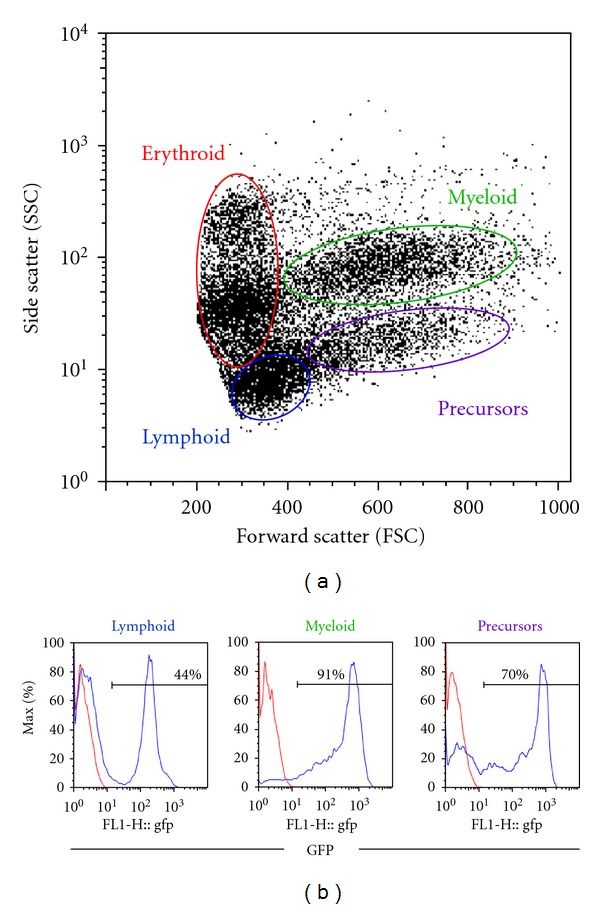
Flow cytometry analysis of zebrafish whole kidney marrow from a marrow transplant recipient. Zebrafish transplant recipients were irradiated and injected with 5 × 10^5^ marrow cells from a transgenic *β-actin:GFP* donor. Whole kidney marrow from a representative recipient was dissected 3 months later and analyzed by flow cytometry. (a) The forward scatter (FSC) versus side scatter (SSC) profile of zebrafish whole kidney marrow shows four cell populations: erythroid, lymphoid, myeloid, and precursor cells. (b) Histograms for GFP expression of cells within the lymphoid, myeloid and precursor gates show multilineage engraftment with GFP^+^ donor cells (blue lines). The red lines show GFP expression in a wild-type-negative control fish.

The first hematopoietic transplant experiments in zebrafish were performed by Traver et al., whose work was published in 2003 [4]. This landmark paper was the first to report the evaluation of zebrafish kidney marrow cells including separation of the major blood cell lineages by flow cytometry, a method which is currently the standard procedure for identifying multilineage engraftment after hematopoietic transplantation in zebrafish (Figure 1(a)). In addition, hematopoietic transplantation was used to rescue two different mutant embryos. The Vlad tepes (*gata*1^−/−^) mutation is homozygous lethal by 14 days after fertilization, and these embryos have a complete absence of erythroid cells [5]. Approximately 100–1000 whole kidney marrow (WKM) cells from a *gata1-GFP* transgenic donor were injected into the circulation of *gata*1^−/−^ zebrafish embryos 48 hours after fertilization (hpf). While untransplanted control embryos did not survive past 14 dpf, 20–60% of the transplant recipients survived long term, up to 8 months after transplant. All surviving recipients had circulating GFP^+^ red blood cells, indistinguishable from the *gata1-GFP* donors [4]. 

Taking these embryonic transplant experiments one step further, donor marrow was isolated from double transgenic *β*-*actin-GFP/gata1-dsRED* fish, in order to monitor donor-derived cells from multiple lineages. The *β*-*actin-GFP* transgene is expressed by almost all zebrafish cell types, including all leukocytes. Erythrocytes do not express *βactin*, so they are marked by the *gata1-dsRED* transgene instead. For these experiments, the transplant recipients were *bloodless (bls)* mutants, a dominant, partially penetrant mutation resulting in absent primitive hematopoiesis, but preserved adult hematopoiesis [6]. Injection of double-positive WKM cells into 48 hpf *bls* mutants allowed independent tracking of GFP^+^ leukocytes and dsRED^+^ erythrocytes in the recipient embryos [4]. Sustained multilineage donor-derived cells were visible in the circulation of transplant recipients at 8 weeks after transplantation, indicating successful engraftment of long-term hematopoietic repopulating cells.

### 2.2. Adult Marrow Cells into Adult Recipients

Following up on their transplantation experiments into embryos, Traver et al. subsequently performed transplantation of WKM cells into adult recipients [7]. After using ionizing radiation as pretransplant conditioning to ablate the recipient's hematopoietic cells, including the immune system, approximately 1 × 10^6^ *β*-*actin-GFP/gata1-dsRED* donor marrow cells were delivered into the recipient's circulation by direct intracardiac injection. When irradiated with 40 Gy, a lethal dose, all the untransplanted animals died by 14 days after irradiation. However, >70% of the animals receiving WKM cells after irradiation were rescued, and survived at least 30 days after irradiation. As in the experiments with embryonic transplant recipients, GFP^+^ leukocytes and dsRED^+^ erythrocytes were visible in the circulation of the engrafted adult recipients using fluorescence light microscopy [7]. FACS analysis of recipient WKM showed robust multilineage engraftment with >86% GFP^+^ cells up to 8 weeks after transplant (Figure 1(b)). 

### 2.3. Embryonic HSCs into Embryos

Similar to murine embryonic HSCs, the first HSCs in the developing zebrafish are located in the aorta-gonad-mesonephros (AGM) [8]. Initial experiments to identify these HSCs in zebrafish relied upon anatomic similarities with murine embryonic HSCs. Cells expressing *cmyb, runx1, *and CD41 are observed in the ventral wall of the dorsal aorta in zebrafish embryos 24–36 hpf [9–12], similar to the expression noted in the ventral wall of the aorta in murine embryos [13]. These *cmyb*+ and *runx1+* cells were presumed to be embryonic definitive HSCs, although functional evaluation of these cells was lacking. Using CD41 as another marker of embryonic HSCs, Bertrand et al. sorted CD41^+^/gata1^−^ donor cells by flow cytometry from *CD41-eGFP/gata1-dsRED* double transgenic embryos at 72 hpf [14]. These cells were then injected into the sinus venosus of age-matched wild-type embryos. Within one day after transplant, donor-derived cells were observed in the caudal hematopoietic tissue (CHT) and thymi of recipients. Although the transplanted donor cells had been dsRED negative, subsequent erythroid differentiation of engrafted cells revealed dsRED^+^ cells in the circulation of recipients [14]. These experiments helped to prove that CD41^+^ cells in the AGM are capable of colonizing definitive hematopoietic organs, namely, the thymus and CHT, in developing zebrafish, and therefore, this population includes the first developing HSCs in the embryo.

### 2.4. A Competitive Transplantation Assay for Chemical Screening

**Figure 2:**
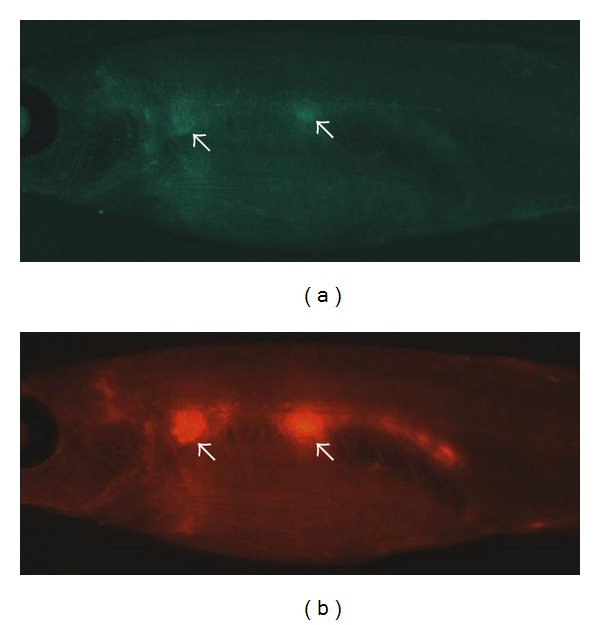
Direct visualization of engrafted GFP^+^ and mCherry^+^ marrow donor cells in *casper* recipients. 40 × 10^3^ WKM cells from a transgenic *ubiquitin:GFP* donor were mixed with 80 × 10^3^ WKM cells from a transgenic *ubiquitin:mCherry* donor and injected into the circulation of a *casper* recipient fish. The photos are taken 4 weeks after transplantation and show engraftment of (a) GFP^+^ and (b) mCherry^+^ cells in the kidney (white arrows).

Capitalizing on the relative ease of *in vivo* chemical screening using the zebrafish model, Li et al. have utilized a competitive hematopoietic transplantation assay to search for chemicals that enhance hematopoietic engraftment (manuscript submitted). Marrow cells from *β*actin-GFP fish were incubated *ex vivo* in chemicals from a panel of more than 2000 known bioactive compounds. After pretreatment, the *β*actin-GFP WKM was mixed at a standard ratio with WKM from commercially available red Glofish, and transplanted into *casper* recipient fish [15]. Normally kidney marrow fluorescence is not visible in an adult animal due to the presence of pigmentation in the skin. However, *casper* fish are homozygous for two pigment mutations, *roy* and *nacre*, and therefore have transparent skin, allowing visualization of engrafted fluorescent marrow cells *in vivo*. Unlike prior studies examining engraftment at a single time point by FACS analysis of multilineage WKM populations, this screen also followed the level of GFP^+^ and RFP^+^ cells in the kidney of anesthetized recipients at several time points after transplant (Figure 2). The ratio of green : red marrow cells by fluorescence microscopy *in vivo* was highly correlated with the green : red ratio measured by flow cytometry of the dissected WKM cell preparation. All chemicals identified in the screen that stimulated enhanced engraftment were also tested in murine transplants to validate the effects in an immune-matched mammalian transplant assay. In total, ten compounds were identified in the screen that resulted in enhanced green : red ratio, and these are currently undergoing further evaluation.

## 3. Importance of Immune Matching ****in Hematopoietic Transplantation

None of the transplantation experiments described to this point took into account any aspect of immunologic matching, as isogenic and congenic fish lines were not available. This fact highlights another significant difference between murine and zebrafish marrow transplants, namely that murine donors and recipients are congenic and hence immunologically identical. In contrast, although many commonly used zebrafish lines (e.g., AB, Tubingen, and wik) have been repeatedly incrossed through decades of laboratory use, attempts to generate truly isogenic or congenic zebrafish lines have largely failed due to inbreeding depression such that these fish lines could no longer be maintained [16]. In addition, sex skewing of clutches, whereby a generation of siblings was all the same sex, has also hindered the ability to maintain highly inbred fish lines. Despite this disadvantage, significant progress has still been made developing hematopoietic transplantation methods in the zebrafish over the past decade, as described above. 

As more sophisticated transplantation experiments are designed to ask more complex questions about stem cell biology, the need for immune matching becomes more critical. When transplanting any allogeneic tissue into an adult recipient with a competent immune system, one would expect a lack of immune matching to result in rejection of the transplanted tissue (reviewed in [17]). In the zebrafish, immune matching is not required in embryonic recipients younger than 5 days after fertilization, as thymic development is not apparent until then [18]. By 4–6 weeks after fertilization, the cellular and humoral immune system is fully functional and would be capable of rejecting any transplanted tissue that was not histocompatible [19, 20]. Pretransplant conditioning with radiation is commonly used to suppress the immune system of adult murine and zebrafish recipients, and in the case of hematopoietic transplants to give the added advantage of clearing the marrow niche. For zebrafish recipients receiving a sublethal dose of radiation, the transplanted tissue is still rejected once the recipient's immune cells recover, approximately 4 weeks after irradiation [21]. 

Another consequence of immune mismatch between transplant donors and recipients occurs uniquely in the setting of hematopoietic transplantation. When engrafted immune cells recognize the recipient as “nonself,” an immune response is mounted against the recipient's tissues resulting in graft-versus-host disease (GVHD), a phenomenon that is also observed clinically in human allogeneic bone marrow transplant [22]. Therefore, the importance of immune matching in hematopoietic transplantation impacts not only initial engraftment, but also the health and survival of the recipient if the engrafted hematopoietic cells attack the host.

## 4. Methods to Quantitate Hematopoietic**** Engraftment

Comparing the function of two HSC populations involves a competitive hematopoietic transplantation assay where both populations engraft in the same transplant recipient (reviewed by Purton and Scadden in [23]). This experimental design is required when mutant marrow cells from one donor are hypothesized to have defective hematopoietic engraftment. The mutant cells are transplanted into the recipient together with a radio-protective dose of wild-type marrow cells. If the mutant HSCs are defective, the wild-type HSCs will out-compete them, and the donor chimerism of the recipient will highly favor the wild-type donor cells. Without these wild-type HSCs to rescue the recipient, lack of engraftment of the mutant cells would likely result in the recipient's death, and there would be no blood or marrow cells to evaluate at the end of the experiment. Using a competitive experimental design ensures that all the recipients survive until the end of the experiment and their data are included in the final analyses. In the event that the mutant marrow has normal HSC function, the donor chimerism would reveal an equal mix of engrafted hematopoietic cells from both donors. Immune matching of both donors and the recipient is an essential component of any competitive hematopoietic transplantation assay. Otherwise, one cannot rule out biased immune rejection of one donor's cells compared to the other, and the engraftment “winner” may merely reflect immunologic differences and not a difference in stem cell biology.

A variation of the competitive hematopoietic transplantation assay is the limit dilution assay. This method is the gold standard for quantitating HSC content and also requires all donors and recipients to be immunologically matched. This assay involves transplantation of serially diluted marrow cells such that fewer and fewer marrow cells are given to subsequent transplant recipients, while a constant number of wild-type marrow cells are given simultaneously to radioprotect the recipients. Engraftment and donor chimerism are evaluated for each recipient, and then Poisson statistics are used to calculate the number of long-term repopulating cells contained in the original marrow population [24]. The ability to perform these competitive and quantitative experiments using zebrafish HSCs will be essential to characterize stem cell mutants and asking questions about HSC biology. Therefore, a better understanding of the histocompatibility genes in the zebrafish is needed so that these assays can be performed with proper immune matching.

## 5. Histocompatibility Antigens in Zebrafish****  Compared with Other Vertebrates

One of the first multimegabase regions of the human genome to be sequenced, the human major histocompatibility complex (MHC) locus, is located on chromosome 6p21.31 and contains over 200 identified genes within a 3.6 × 10^6^ basepair span [25]. The classical class I and class II genes within the MHC region are the central cell surface proteins responsible for determining tissue histocompatibility of an allograft. This gene-dense region also contains a number of other genes important for the immune response, including antigen-processing genes such as proteasome subunit *β* type (PSMB), complement genes, and the peptide transporters TAP1 and TAP2 [26, 27].

Class I MHC molecules are polymorphic transmembrane proteins with three immunoglobulin-like domains that are expressed on virtually all cell types. They bind noncovalently to *β*2-microglobulin and present endogenously derived peptides to CD8^+^ T lymphocytes (reviewed in [28]). Although class I and II proteins share a similar three-dimensional structure, class II MHC molecules are heterodimeric complexes consisting of an alpha chain and a beta chain, with each chain containing two immunoglobulin-like domains. They present lysosomally derived peptide antigens to CD4^+^ T lymphocytes, and their expression is limited to B-lymphocytes, macrophages, and other antigen-presenting cells.

While most jawed vertebrate species possess linked class I and II genes located within a single chromosomal locus similar to the human MHC, the bony fishes are unique in that they have class I and II genes located on distinct chromosomes [29]. In the zebrafish, at least three relevant loci have been identified. Chromosome 19 contains class I genes as well as some antigen-processing genes, making the locus syntenic to the human MHC locus [30, 31]. However, there are no class II genes on chromosome 19. Instead the zebrafish class II alpha and beta genes are located on chromosome 8 [26, 32]. Chromosome 1 contains additional class I genes, termed “ze” genes, which appear most similar to mammalian nonclassical Class I genes [33]. Finally, the “L” genes, class I genes unique to teleost fish, are located on chromosomes 3 and 8, although they are less polymorphic than other class I genes, and their precise function is not clear [34]. While DNA sequence analyses of the zebrafish MHC genes show similarities with MHC genes of many species, virtually no data are available to evaluate the function or even the cell-surface expression of the class I and II genes in zebrafish. Prior to the transplantation experiments described below, no functional evaluation of any zebrafish MHC genes had been performed. 

## 6. Immune-Matched Hematopoietic ****Transplants in Zebrafish

Following up on the adult marrow transplant experiments published in 2004 [7], subsequent adult transplantation experiments sought to evaluate long-term hematopoietic engraftment greater than 12 weeks after transplant. Having observed poor survival in random donor long-term hematopoietic transplantation experiments (J. L. O. de Jong and L. I. Zon, unpublished data), immune typing of the zebrafish MHC genes was a logical step to ensure that graft rejection and/or GVHD were not contributing to the recipient mortality. In these first hematopoietic transplant experiments with immune matching, the class I MHC genes at the chromosome 19 locus were typed for all the sibling progeny of a single mating pair [35]. Genotyping was achieved by preparing DNA from fin clips of individual fish, then using a panel of PCR primers to amplify MHC gene sequences. The amplified fragments were then sequenced to identify the specific MHC genes present in each individual animal. As expected, there were four MHC haplotypes represented within this family, and approximately 25% of the progeny fell into each of the four genotypes. WKM cells from *β*-actin-GFP^+^ donor fish of each MHC genotype were transplanted into GFP-negative siblings of the same MHC genotype and also into unrelated wild-type recipients, presumed to be mismatched. Survival and donor chimerism were significantly improved in the matched recipients compared with the unmatched recipients (Table 1), indicating the importance of immune matching at the chromosome 19 MHC locus for hematopoietic engraftment [35]. These experiments were the first functional evaluation of any zebrafish MHC genes in a transplantation assay. 

These first experiments did not specifically type for class II genes located on chromosome 8, or other class I genes on other chromosomes. It may be that coincidental matching at the class II locus occurred for a significant number of the related “matched” recipients in these experiments, thereby contributing to improved donor chimerism. 

We expected that immune matching at the class II locus would also be important for hematopoietic engraftment. Therefore, we performed additional transplantation experiments matching the donors and recipients at three separate loci: the two class I loci on chromosomes 1 and 19 and the class II locus on chromosome 8. 2.5 × 10^5^ WKM cells from *β*-actin-GFP^+^ donor fish were transplanted into both completely matched recipients and unmatched, unrelated recipients. Long-term engraftment at 3 months after transplant showed similar donor chimerism results as the transplant experiments with matching at only the chromosome 19 locus (Table 1). These data suggest that matching of the class I genes at the chromosome 19 locus is the most important for tissue histocompatibility in a transplantation assay, and that the additional MHC loci on chromosomes 1 and 8 play a minimal role. Further experiments are underway to individually test the class I genes on chromosome 1 and the class II genes on chromosome 8 to determine the contribution, if any, of these loci to histocompatibility in tissue transplantation.

## 7. Optimizing Survival of Hematopoietic ****Transplant Recipients

Survival of zebrafish hematopoietic transplant recipients is often difficult to predict from one experiment to the next. We have implemented a number of changes to the initially published transplantation protocol to address the problem of poor survival after transplant. While lack of histocompatibility may play a role for some animals, a number of other factors also appear to be important. In our experience, younger fish have better survival than older fish, and optimal recipients are approximately 3-4 months of age (J. L. O. de Jong and L. I. Zon, unpublished data). This may be due to colonization of older fish with bacterial or fungal pathogens that overwhelm and kill the immune-compromised host after transplantation. Maintaining excellent water quality is also critically important to recipient survival. We hypothesized that treatment with prophylactic antibiotics for a few days immediately after transplant might improve survival. However, placing transplant recipients “off system” in fish water containing antibiotics paradoxically caused decreased survival, as fish being treated in this way suffered from quickly deteriorating water quality and high ammonia levels (C. Lawrence, personal communication). While it is impractical to keep a therapeutic level of antibiotics in the large volume of water circulating through an entire aquatic system, the ability to maintain water quality at a consistently high standard resulted in improved survival of our transplant recipients, even without antibiotics.

**Figure 3:**
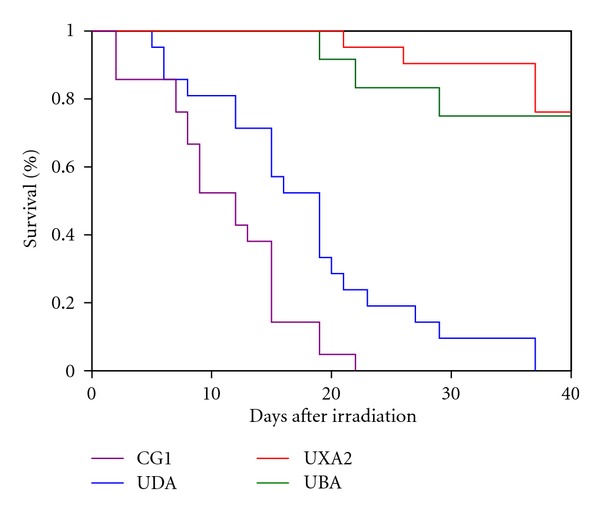
Survival of different zebrafish lines in response to radiation. Kaplan-Meier survival curves are shown for four different zebrafish strains after irradiation with a total dose of 25 Gy, delivered in two equal fractions of 12.5 Gy separated by 24 hours. Twenty one fish were irradiated in each group. CG1 is a clonal homozygous diploid fish line generated by parthenogenesis [21, 38]. UDA, UXA2, and UBA are inbred zebrafish lines derived from a single mating pair of AB parents [35]. Each line was named for the homozygous class I MHC gene at its chromosome 19 locus. The results demonstrate 100% mortality for the CG1 fish by day 22, and by day 37 for the UDA fish. In contrast, the UBA and UXA2 fish lines both had approximately 80% survival at 40 days after irradiation.

Determining the appropriate radiation dose for pretransplant conditioning of recipient fish has also proven more challenging than initially anticipated. Water can greatly attenuate the radiation dose over a short distance. For example, at a depth of 1 cm of water, we have observed that the radiation dose at the bottom of the dish is decreased by about 10–15% compared with the radiation dose at the surface of the water (J. L. O. de Jong, unpublished data). Therefore, it is critically important that fish be placed in a minimal volume and depth of water to ensure that all recipients receive an equivalent radiation dose. The minimum lethal dose of radiation for zebrafish was first reported to be 40 Gy [7]. However, subsequent work showed that this dose was not optimal for pretransplant conditioning, as the mortality of fish was 100%, even after receiving a radio-protective dose of WKM cells. A sublethal dose of 25 Gy provided for maximal survival with engraftment, so this was the dose selected for most experiments [35]. This result suggests that while the hematopoietic compartment is the most radiation-sensitive tissue in the zebrafish, as in mammals, there is a narrow therapeutic index for lethal radiation damage to other tissues. To minimize the radiation injury to nonhematopoietic tissues, many protocols for murine and human bone marrow transplants utilize fractionated radiation dosing. We have now initiated a standard conditioning protocol of 30 Gy split into two equal fractions of 15 Gy, where the two fractions are given 24 hours apart. The survival of these recipients is comparable to animals receiving 25 Gy as a single dose (J. L. O. de Jong, unpublished data). Finally, we have observed that different fish lines have varying sensitivities to radiation. For example, when comparing fish from the AB strain that have been bred to homozygosity at the MHC loci, some were significantly more sensitive to a given radiation dose than others (Figure 3). This result suggests that a radiation dose-response titration should be performed for each strain of recipients to be transplanted in order to determine the optimal radiation dose. Alternatively, conditioning with chemotherapeutic medications such as cyclophosphamide [36] could be used, although these have not been tested for pretransplant conditioning of zebrafish donors.

## 8. Future Directions for Hematopoietic ****Transplantation in the Zebrafish

Although HSC transplantation is a commonly used treatment modality for human diseases, including many malignancies, blood disorders, and immune deficiencies, this procedure continues to have high morbidity and mortality. Difficulties include selecting an optimally matched allogeneic donor, prolonged immune suppression with susceptibility to deadly infections, delayed and/or incomplete immune reconstitution, and maximizing the graft-versus-tumor effect while minimizing graft-versus-host disease. A zebrafish model for hematopoietic transplantation permitting *in vivo* investigation of these challenges would provide a basis to understand the biological mechanisms involved and identify possible solutions to address them. 

### 8.1. Parthenogenesis to Develop Homozygous Diploid Fish Lines

The lack of isogenic and congenic fish lines is a serious handicap for future transplantation experiments with zebrafish. To overcome this barrier, gynogenetic fish lines have been utilized in recent years to successfully transplant liver tumors, acute lymphoblastic leukemia cells, and rhabdomyosarcoma tumor cells into unirradiated immunologically identical adult recipients [21, 37]. Developing these homozygous diploid clonal fish lines is labor intensive, time consuming and inefficient [38, 39]. However, once a robust line is generated, it can be used to make transgenic donors with fluorochrome-labeled marrow cells. These donors could then be used to perform competitive HSC transplants using immunologically identical donors and recipients. Developing a homozygous diploid fish line from *casper* fish would be even more useful, as the advantages of analyzing engraftment at many time points could also be realized in the setting of an immune-matched competitive transplant. Efforts are currently underway to generate these fish.

### 8.2. Minor Histocompatibility Antigens

Further work will also be valuable to identify all the specific class I and II genes important for histocompatibility in the zebrafish, both for a basic understanding of zebrafish immunology, as well as the implications for optimizing future transplant experiments. When a zebrafish mutant has a postulated HSC defect, scientists need to have immune-matched recipients to test whether marrow cells from the mutant zebrafish have flawed engraftment in a competitive transplantation assay. Without immune matching, such an assay will be difficult to interpret. The ability to immunotype any random zebrafish, and thereby select appropriately matched donors and recipients would allow for a much quicker time frame to perform these experiments, compared with generations of inbreeding, which may be unsuccessful given the history of prior attempts to generate such inbred zebrafish lines. However, even having a donor with “perfect” matching at the MHC locus, human bone marrow transplant recipients are still at risk for GVHD, likely due to mismatched minor histocompatibility antigens on other chromosomes. Therefore, identifying both major and minor histocompatibility antigens throughout the genomes that are relevant for transplant rejection and GVHD in the zebrafish will be critical to prospectively determine optimally matched donors and recipients. This information will clearly be useful for zebrafish experiments, as described above. In addition, identifying significant minor histocompatibility antigens in the zebrafish would suggest minor histocompatibility antigens that may also be relevant for human bone marrow transplantation and GVHD. Such work may impact the selection of human bone marrow transplant donors to minimize this potentially devastating outcome after human BMT.

### 8.3. Developing a Zebrafish Model for GVHD

Finally, in the process of fully characterizing the zebrafish histocompatibility genes, we expect to identify recipients with GVHD. To date, we have observed transplant recipients that develop severe edema and ascites resulting in flaring of their scales. This condition in the zebrafish is generically termed “dropsy” and likely can result from a myriad of causes. We postulate that in the setting of hematopoietic transplantation, some of these recipient fish may have GVHD, although further work is needed to fully characterize the “dropsy” phenotype after transplant and confirm the pathophysiology of this diagnosis. By characterizing the GVHD phenotype in zebrafish and developing a zebrafish model of GVHD, one could exploit the advantages of genetic and small molecule-based screening to further characterize the pathways that regulate GVHD. Such experiments may discern mechanisms to minimize GVHD while maximizing the graft-versus-leukemia effect in bone marrow transplant patients.

## 9. Conclusion

As a model for human disease, the zebrafish holds numerous advantages. Gaining knowledge of the functional Class I and II genes in the zebrafish will enhance our understanding of basic zebrafish biology, as well as the ability to use this versatile animal model to ask questions about tissue transplantation, including hematopoietic stem cells, other normal tissues and cancers cells. This work will likely inform mammalian biology, improving our understanding of human HSCs, and has the potential to impact the treatment of patients undergoing bone marrow transplantation.

## Figures and Tables

**Table 1:** Mean percentage of GFP^+^ cells in engrafted recipient zebrafish receiving MHC-matched or -unmatched donor marrow.

	Only Chr 19 matched [35]		Chr 1, 8, 19 all matched	
Myeloid matched	47.86 ± 30.9	*P* = 0.0002	52.36 ± 25.43	*P* = 0.0036
Myeloid unmatched	6.45 ± 1.77		11.58 ± 7.03	

Lymphoid matched	10.51 ± 19.88	*P* = 0.05	9.51 ± 12.32	*P* = 0.047
Lymphoid unmatched	1.28 ± 0.38		3.47 ± 4.601	

Data are mean ± S.D.
